# Mental health outcomes in patients with inherited retinal diseases: a systematic review and meta-analysis

**DOI:** 10.1186/s40942-026-00820-7

**Published:** 2026-02-20

**Authors:** Hashem Abu Serhan, Abdullah Ahmed, Oase Sbei, Waleed Kojan, Mahrukh Chaudhry, Mubashra Khalid, Ahmed Sheraz, Ahmad Al-Moujahed

**Affiliations:** 1 Department of Ophthalmology, Hamad Medical Corporation, Doha, Qatar; 2 University of Illinois, Chicago, USA; 3 Wayne State University School of Medicine, Detroit, MI USA; 4 The University of Toledo College of Medicine and Life Sciences, Toledo, OH USA; 5 Allama Iqbal Medical College, Lahore, Pakistan; 6 Department of Ophthalmology, Byers Eye Institute, Stanford University School of Medicine, Palo Alto, CA USA

**Keywords:** Inherited retinal diseases, Retinitis pigmentosa, Stargardt disease, Depression, Anxiety, Mental health

## Abstract

**Purpose:**

To assess the association between inherited retinal diseases (IRDs) and mental health outcomes, specifically examining the prevalence of depression and anxiety compared to general population estimates.

**Methods:**

Following PRISMA 2020 guidelines, we conducted a search of PubMed/MEDLINE, Scopus, Ovid, and Web of Science from inception through April 2025. Studies reporting quantitative prevalence data for depression and/or anxiety in IRD patients using validated instruments or standardized diagnostic criteria were included. Random-effects meta-analyses using the DerSimonian-Laird method with Freeman-Tukey double arcsine transformation were performed. Heterogeneity was assessed using I² statistics, and methodological quality was evaluated using the Newcastle-Ottawa Scale. Subgroup analyses examined prevalence by disease type, and sensitivity analyses tested result robustness.

**Results:**

Sixteen studies encompassing 12,868 participants met inclusion criteria. The pooled depression prevalence was 31.0% (95% CI: 22.1%-40.6%; I² = 93.9%), substantially exceeding general population rates. Anxiety prevalence was 29.3% (95% CI: 17.1%-43.3%; I² = 94.3%). Subgroup analysis revealed depression prevalence of 30.6% (95% CI: 22.4%-39.4%) for Retinitis Pigmentosa and 42.5% (95% CI: 0.0%-97.7%) for Stargardt disease. Other IRDs were represented by a single study reporting 14.6% prevalence. The test for subgroup differences approached statistical significance (*p* = 0.08). Sensitivity analyses confirmed result robustness despite substantial heterogeneity. Publication bias assessment suggested potential overestimation of depression prevalence.

**Conclusion:**

Patients with IRDs experience a substantial mental health burden, with depression and anxiety prevalence markedly exceeding general population rates. These findings underscore the critical need for mental health screening integrated into routine ophthalmologic care.

**Supplementary Information:**

The online version contains supplementary material available at 10.1186/s40942-026-00820-7.

## Introduction

Inherited retinal diseases (IRDs) represent a diverse group of genetically determined disorders affecting retinal structure and function, often leading to progressive visual impairment and blindness. Among these conditions, retinitis pigmentosa (RP) and Stargardt disease are two of the most common and extensively characterized entities. Retinitis pigmentosa, comprising a group of rod-cone dystrophies, manifests as progressive peripheral vision loss and night blindness, typically emerging in adolescence or early adulthood and ultimately advancing to central vision loss [1]. Stargardt disease, the most prevalent form of inherited macular degeneration in children and young adults, presents with central vision loss resulting from lipofuscin accumulation in the retinal pigment epithelium [2].

The impact of IRDs extends far beyond the physical manifestations of vision loss, imposing a profound psychosocial and emotional burden on affected individuals. Visual impairment has been consistently linked to elevated risk for mental health disorders, particularly depression and anxiety [3–5]. However, individuals with IRDs face unique psychological challenges stemming from early disease onset, chronic progressive deterioration, and the hereditary nature of their condition, which frequently affects multiple family members [6]. The unpredictability of vision loss trajectory, increasing dependence on caregivers, barriers to education and employment, and progressive social isolation constitute commonly reported stressors in this vulnerable population [7].

Emerging evidence highlights the substantial mental health burden associated with IRDs. One large-scale study demonstrated that patients with RP are five to six times more likely to develop symptoms of depression or anxiety compared to the general population [7]. Additional research has documented markedly reduced quality of life among IRD patients, particularly affecting domains of independent living, sensory function, and interpersonal relationships [7]. Furthermore, qualitative investigations utilizing semi-structured interviews have revealed that Stargardt disease significantly compromises patients’ mental health, with prevalent reports of frustration, persistent worry, and impaired concentration, alongside broader impairments in physical, social, and role functioning [8]. Despite these important findings, the literature remains fragmented, with insufficient quantification of the true prevalence and severity of psychiatric comorbidities in IRD populations.

Critical gaps persist in the existing literature. Mental health prevalence data often lack appropriate comparison groups, and few studies directly contrast psychiatric outcomes between IRD patients and individuals without vision loss. The complex interplay between visual disability, social determinants of health, genetic counseling needs, and mental health support requirements is rarely addressed comprehensively in either ophthalmologic or psychiatric care settings. This fragmentation contributes to systematic underdiagnosis and inadequate treatment of mental illness in visually impaired populations [9].

This meta-analysis aims to systematically assess the association between IRDs, specifically RP and Stargardt disease, and mental health outcomes, focusing on the prevalence of depression and anxiety. By comparing affected individuals to the general population or those without inherited retinal disorders, we seek to generate robust estimates of mental health burden, identify trends across age groups and diagnostic categories, and provide evidence-based recommendations for integrated mental health support in this vulnerable population.

## Methods

### Protocol and registration

This systematic review and meta-analysis was conducted in accordance with the Preferred Reporting Items for Systematic Reviews and Meta-Analyses (PRISMA) 2020 guidelines [10]. The protocol was prospectively registered with PROSPERO [CRD420251175340].

### Search strategy

A comprehensive systematic literature search was conducted across four electronic databases: PubMed/MEDLINE, Scopus, Ovid and Web of Science from inception through April 2025. The search strategy employed a combination of Medical Subject Headings (MeSH) terms and free-text keywords related to three core concepts: (1) inherited retinal diseases (“retinitis pigmentosa,” “rod-cone dystrophy,” “Stargardt disease,” “STGD1,” “fundus flavimaculatus,” “inherited retinal disease,” “inherited retinal dystrophy,” “hereditary retinal degeneration”); (2) mental health outcomes (“depression,” “depressive disorder,” “major depressive disorder,” “anxiety,” “anxiety disorder,” “generalized anxiety,” “mental health,” “psychological distress,” “psychiatric disorder,” “suicidal ideation”); and (3) epidemiological terms (“prevalence,” “incidence,” “epidemiology,” “frequency”). Search terms were adapted for each database using appropriate syntax and controlled vocabulary. No language or publication date restrictions were applied during the initial search. Detailed search criteria is given in Supplementary Table S1.

### Inclusion and exclusion criteria

Two independent reviewers screened all titles and abstracts against predetermined eligibility criteria. Studies were included if they: (1) involved patients with genetically or clinically diagnosed inherited retinal diseases, including RP, Stargardt disease, or other IRDs; (2) reported quantitative prevalence data for depression and/or anxiety based on standardized diagnostic criteria (DSM-IV/5, ICD-9/10) or validated psychometric instruments (e.g., Beck Depression Inventory, Hospital Anxiety and Depression Scale, Patient Health Questionnaire-9, Generalized Anxiety Disorder-7); and (3) provided sufficient numerical data to calculate proportions (number of cases and total sample size).

Studies were excluded if they: (1) were case reports, reviews, meta-analyses, editorials, conference abstracts, or qualitative studies without quantitative prevalence data; (2) lacked extractable prevalence data or clear diagnostic thresholds; (3) focused exclusively on acquired or non-inherited retinal diseases, or included mixed populations where IRD patients could not be analyzed separately; (4) enrolled patients with significant comorbid neurological or psychiatric conditions that preceded the IRD diagnosis; or (5) reported only general psychological distress without specific depression or anxiety measurements using validated instruments. Disagreements between reviewers during screening and eligibility assessment were resolved through discussion and consultation with a third reviewer when necessary. The complete study selection process is detailed in the Results section.

### Data extraction

Data extraction was performed independently by two investigators using a standardized electronic data extraction form piloted on three studies prior to full implementation. Discrepancies were resolved through consensus discussion or arbitration by a third reviewer. For each included study, the following information was systematically extracted: first author, publication year, country of origin, study design, sample size, demographic characteristics (mean age, age range, gender distribution), IRD diagnosis and subtype (retinitis pigmentosa, Stargardt disease, other IRDs), diagnostic confirmation method, depression and anxiety assessment tools, diagnostic criteria or cutoff scores used, number of participants meeting criteria for depression and/or anxiety, and prevalence rates with confidence intervals when reported.

For studies reporting data on multiple IRD subtypes, subgroup-specific data were extracted separately when available. When studies used multiple assessment instruments or reported results at different cutoff thresholds, we extracted data from the validated instrument most commonly used across studies or the threshold most consistent with clinical diagnostic criteria. Authors of studies with unclear or incomplete data were contacted via email for clarification; if no response was received after two contact attempts over four weeks, the study was excluded from analysis if data could not be reliably extracted.

### Quality assessment

Methodological quality of included studies was assessed independently by two reviewers using an adapted version of the Newcastle-Ottawa Scale (NOS) for cross-sectional and cohort studies [11]. The NOS evaluates three broad domains: selection of study groups (representativeness of the sample, ascertainment of exposure, selection of controls), comparability of groups (control for confounding factors), and outcome assessment (assessment method, follow-up adequacy). Each study was assigned a quality score based on a star rating system, with a maximum of 9 stars. Studies scoring ≥ 7 stars were considered high quality, 4–6 stars moderate quality, and < 4 stars low quality. Studies were not excluded based on quality scores; instead, quality assessment informed sensitivity analyses and interpretation of findings. Disagreements in quality ratings were resolved through discussion.

Quality assessment revealed that 9 studies (53%) were rated as high quality (7–9 stars), including three studies achieving the maximum score of 9 stars [12–14], and six studies scoring 7–8 stars [7, 15–19]. Seven studies (41%) were classified as moderate quality (4–6 stars) [20–26]. The most common methodological strengths across studies included clear case definitions and validated outcome assessment instruments. Primary limitations included lack of matched control groups in cross-sectional studies, convenience sampling methods, and insufficient adjustment for potential confounders (Supplementary Table S2).

### Statistical analysis

Meta-analyses were conducted separately for depression and anxiety prevalence using R statistical software (version 4.3.0) with the *meta* package (version 6.5-0). Due to anticipated clinical and methodological heterogeneity across studies, a random-effects model using the DerSimonian-Laird method was employed to calculate pooled prevalence estimates with 95% confidence intervals (CIs). Individual study prevalences were transformed using the Freeman-Tukey double arcsine transformation to stabilize variances, particularly for studies with prevalences near 0% or 100%, and back-transformed for presentation of pooled estimates.

Between-study heterogeneity was quantified using Cochran’s Q statistic (with statistical significance set at *p* < 0.10) and the I² statistic, which represents the percentage of total variation across studies attributable to heterogeneity rather than chance. I² values of 25%, 50%, and 75% were interpreted as low, moderate, and high heterogeneity, respectively. The between-study variance (τ²) was also calculated and reported. Subgroup analyses were conducted based on IRD subtype (retinitis pigmentosa and related conditions, Stargardt disease, other IRDs) to explore sources of heterogeneity. The test for subgroup differences was performed using a mixed-effects model, with statistical significance set at *p* < 0.05. Additional exploratory subgroup analyses examined differences in prevalence by assessment methodology when sufficient studies were available.

Sensitivity analyses were performed using the leave-one-out method, whereby the meta-analysis was repeated sequentially with each study omitted once to assess the influence of individual studies on the pooled estimate and to evaluate robustness of findings. Changes in pooled prevalence, confidence intervals, and heterogeneity statistics (I² and τ²) were examined for each iteration. Publication bias was assessed visually using funnel plots and statistically using Egger’s linear regression test for the depression meta-analysis. Due to the limited number of studies in the anxiety meta-analysis (*n* = 3), formal publication bias assessment was not performed, as these tests require a minimum of 10 studies for adequate statistical power. Statistical significance for publication bias was set at *p* < 0.10.

All statistical tests were two-sided, with p-values < 0.05 considered statistically significant unless otherwise specified. Results are presented in accordance with PRISMA guidelines, with forest plots displaying individual study prevalences, pooled estimates, 95% confidence intervals, and heterogeneity statistics.

## Results

### Study Selection

**Fig. 1:**
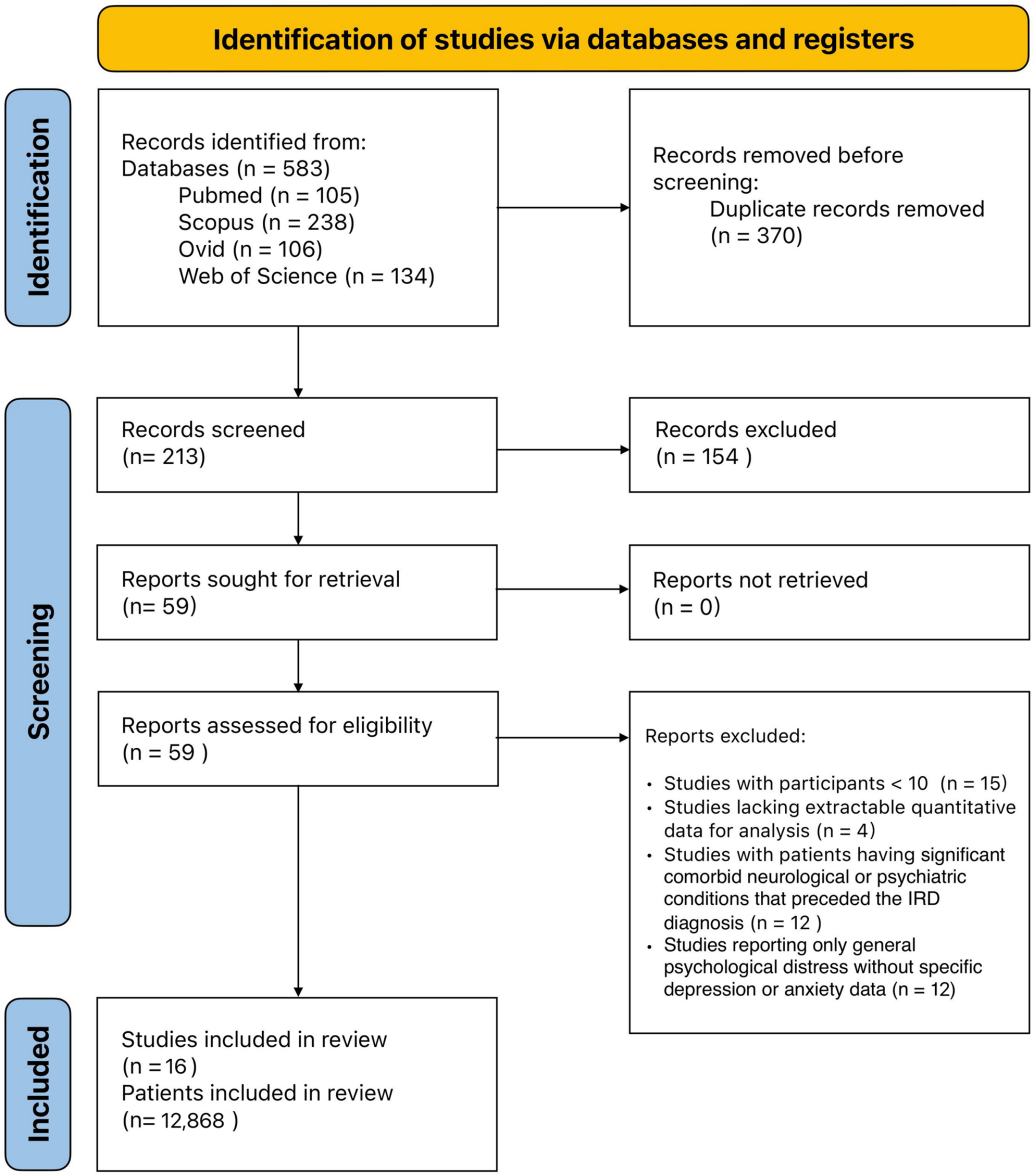
PRISMA flow diagram of study selection process

The systematic search across four electronic databases yielded 583 records: PubMed/MEDLINE (*n* = 105), Scopus (*n* = 238), Ovid (*n* = 106), Web of Science (*n* = 134). (Fig. 1**)** Following removal of 370 duplicate records using reference management software, 213 unique records underwent title and abstract screening by two independent reviewers.

After initial screening, 154 records were excluded based on title and abstract review, leaving 59 reports for full-text assessment. All 59 full-text articles were successfully retrieved (*n* = 0 not retrieved) and assessed for eligibility against predetermined inclusion and exclusion criteria. During full-text assessment, 43 additional studies were excluded for the following reasons: studies with fewer than 10 participants (*n* = 15), studies lacking extractable quantitative data for meta-analysis (*n* = 4), studies enrolling patients with significant comorbid neurological or psychiatric conditions that preceded the IRD diagnosis (*n* = 12), and studies reporting only general psychological distress without specific depression or anxiety data using validated instruments (*n* = 12). Ultimately, 16 studies met the inclusion criteria and were included in the final analysis.

### Baseline characteristics of included studies

The systematic search identified 16 studies eligible for depression meta-analysis, encompassing 12,868 participants with inherited retinal disorders across multiple countries and assessment methodologies. The included studies were predominantly cross-sectional in design (*n* = 13), with three retrospective studies [7, 16, 21]. Studies originated from diverse geographical regions including Iran [20], France [15, 23], United States [7, 21], Spain [22], Brazil [12], Portugal [27], Korea [13, 16, 19], Greece [14, 18], Japan [25], and others [19, 26, 28]. Sample sizes varied considerably, ranging from 27 participants in the smallest study [21] to 10,879 participants in the largest population-based cohort [16]. The mean age of participants across studies ranged from 36.9 ± 5.4 years ^18^ to 60.7 ± 15.4 years [25], reflecting diverse patient populations at different disease stages. Gender distribution showed slight female predominance overall, with individual studies reporting female proportions ranging from 33% [13] to 70% [12].

The majority of studies focused on retinitis pigmentosa and related conditions (*n* = 13), while two studies specifically examined Stargardt disease [12, 18] and one [14] investigated mixed inherited retinal disorder populations (Yioti 2017 [14] included 48 patients with various IRDs: retinitis pigmentosa (*n* = 17, 35.4%), cone-rod dystrophy (*n* = 10, 20.8%), Stargardt’s disease (*n* = 10, 20.8%), Best’s disease (*n* = 3, 6.3%), Usher syndrome (*n* = 3, 6.3%), cone dystrophy (*n* = 3, 6.3%), and pattern dystrophy (*n* = 2, 4.2%). Assessment methodologies for depression varied substantially, including standardized instruments such as the Beck Depression Inventory (BDI) [17, 19, 21, 22], Hospital Anxiety and Depression Scale (HADS) [15, 23, 25]. Patient Health Questionnaire-9 (PHQ-9) [14, 18], and administrative diagnostic codes [7, 16].

Several studies required data adjustments during analysis. Four duplicate entries were identified and removed from the initial dataset to prevent double-counting of participants. For the Öner et al. (2024) study [19], we extracted only moderate-to-severe depression cases (Beck Depression Inventory [BDI] score ≥ 20) rather than all depression levels including mild symptoms. This methodological decision maintained consistency with clinically significant thresholds employed across other included studies. The original study reported 67 cases (90.5%) when including mild depression (BDI 14–19); our extraction yielded 45 cases (60.8%) using the moderate-to-severe threshold, aligning with the BDI ≥ 20 cutoff used in most depression research. For anxiety analysis, only 3 studies [7, 23, 25] provided adequate prevalence data from a total of 950 participants. The López-Justicia 2011 study [17] was excluded from anxiety meta-analysis as it only reported group means without prevalence data, and the reported mean anxiety scores were below clinical cutoff thresholds. The detailed characteristics of all included studies is given in Table 1.

**Table 1:** Baseline Characteristics of Included Studies

Study	Year	Country	Study Design	Sample Size (Total)	Sample Size (Patient)	Sample Size (Control)	Type of Retinal Disorder	Time Period	Age Patient Group (Mean ± SD)	Gender Patient (M/F)	Disease Duration (Mean ± SD)	Age at Diagnosis	% Family History
Adhami-Moghadam 2014	2014	Iran	Cross-sectional	417	417	-	Retinitis Pigmentosa (RP)	January 2009-January 2010	Not specified (broken down into age groups)	231/186	At least 1 year	Not reported, but includes onset from infancy to adulthood	24.20%
Azoulay 2015	2015	France	Cross-sectional	80	60	20	Retinitis Pigmentosa (RP)	Not explicitly stated	44 ± 14	N/A	Not reported	Not reported	Not reported
Bittner et al. 2011	2011	United States	Retrospective observational	27	27	-	Retinitis Pigmentosa (RP)	December 2008 to April 2010	51, range 20–76 years	14/13	-	-	-
Chacón-López 2016	2016	Spain	Cross-sectional observational	51	51	-	Retinitis Pigmentosa (RP)	Not explicitly stated (approx. 2015–2016)	41.85 ± 12.03	15/36	14.35 ± 10.33 years	Not reported	Not reported
Chaumet-Riffaud 2017	2017	France	Cross-sectional observational	148	148	-	Retinitis Pigmentosa (non-syndromic and syndromic, including Usher syndrome type 2)	April 2013-October 2015	38.2 ± 14.7 years	70/78	Median: 10.2 years (range: 0–40)	Median: 24.6 years (range: 3–50)	16.2% had syndromic RP
Gomes 2020	2020	Brazil	Observational cross-sectional	87	41	46	Stargardt’s Disease	Jan 2016-Sep 2017	37.5 ± 13.7	13/28	19.6 ± 9.8 years (living with low vision)	13.0 ± 8.0	-
Humphries 2024	2024	Florida	Cross-sectional survey questionnaire	38	38	-	Retinitis Pigmentosa (RP)	June 2021 to February 2022	50.8 ± 21.9	10/28	-	< 18 years: 30/38 (78.9%), 18–24: 1/38 (2.6%), 25–34: 2/38 (5.3%), 35–44: 3/38 (7.9%), 45–54: 2/38 (5.3%)	22/38 (57.9%) family history; 15/22 (68.2%) immediate family
Kim 2013	2013	Korea	Cross-sectional observational case-control	374	187	187	Retinitis Pigmentosa (RP)	December 2010-February 2011. Control group: 2007–2009	40.1 ± 11.0	125/62	Classified as < 1 year or > 1 year	Not reported	Not reported
Kim 2024	2024	Korea	Retrospective nationwide population-based cohort	10,879	10,879	-	Retinitis Pigmentosa (RP)	2011–2021 (analysis: October 2023-January 2024)	Not reported (age groups: <20: 638; 20–39: 2233; ≥40: 8008)	5169/5710	-	< 20: 638 (5.9%), 20–39: 2233 (20.5%), ≥ 40: 8008 (73.6%)	Not reported
Le 2021	2021	North Carolina	Retrospective case-control	6,093,833	690	6,093,143	Retinitis Pigmentosa (RP)	July 1, 2004-August 30, 2019	Not reported by group; overall: <18: 12.8%, 18–64: 59.1%, 65+: 29.2%	Data for total population: 2,818,738 M / 3,275,095 F	-	-	-
López-Justicia 2010	2010	Spain	Observational cross-sectional	88	32	34 (plus 22 family members)	Retinitis Pigmentosa (RP)	2010	42.5 ± 11.73	4/28	-	-	-
Moschos 2015	2015	Greece	Case-control	69	34	35	Stargardt Disease	N/A	48.7 ± 14.9 (RP patients, not Stargardt)	14/20	Not reported	Not reported	Not reported
Öner 2024	2024	Turkey	Cross-sectional	134	74	60	Retinitis Pigmentosa (RP)	January-December 2023	39.20 ± 12.4	40/34	Not reported	Not reported	75% parental consanguinity
Sainohira 2018	2018	Japan	Prospective cross-sectional	112	112	-	Retinitis Pigmentosa (RP)	August 2015-February 2017	60.7 ± 15.4	46/66	Not reported	Not reported	Not reported
Tamayo 1996	1996	Colombia	Observational cross-sectional	30	30	-	Retinitis Pigmentosa associated with Usher Syndrome	-	Range 3–42 years	15/15	-	-	9 families had > 1 affected child
Yioti 2017	2017	Greece	Cross-sectional observational	144	48	96	Inherited Retinal Dystrophies (IRD): RP, cone-rod dystrophy, Stargardt’s, Best’s, Usher syndrome, cone dystrophy, pattern dystrophy	2014–2015	39.8 ± 15.7	26/22	18.2 ± 15.63 years	Not reported	60.4% reported genetic/hereditary cause

### Outcomes

#### Prevalence of depression

**Fig. 2:**
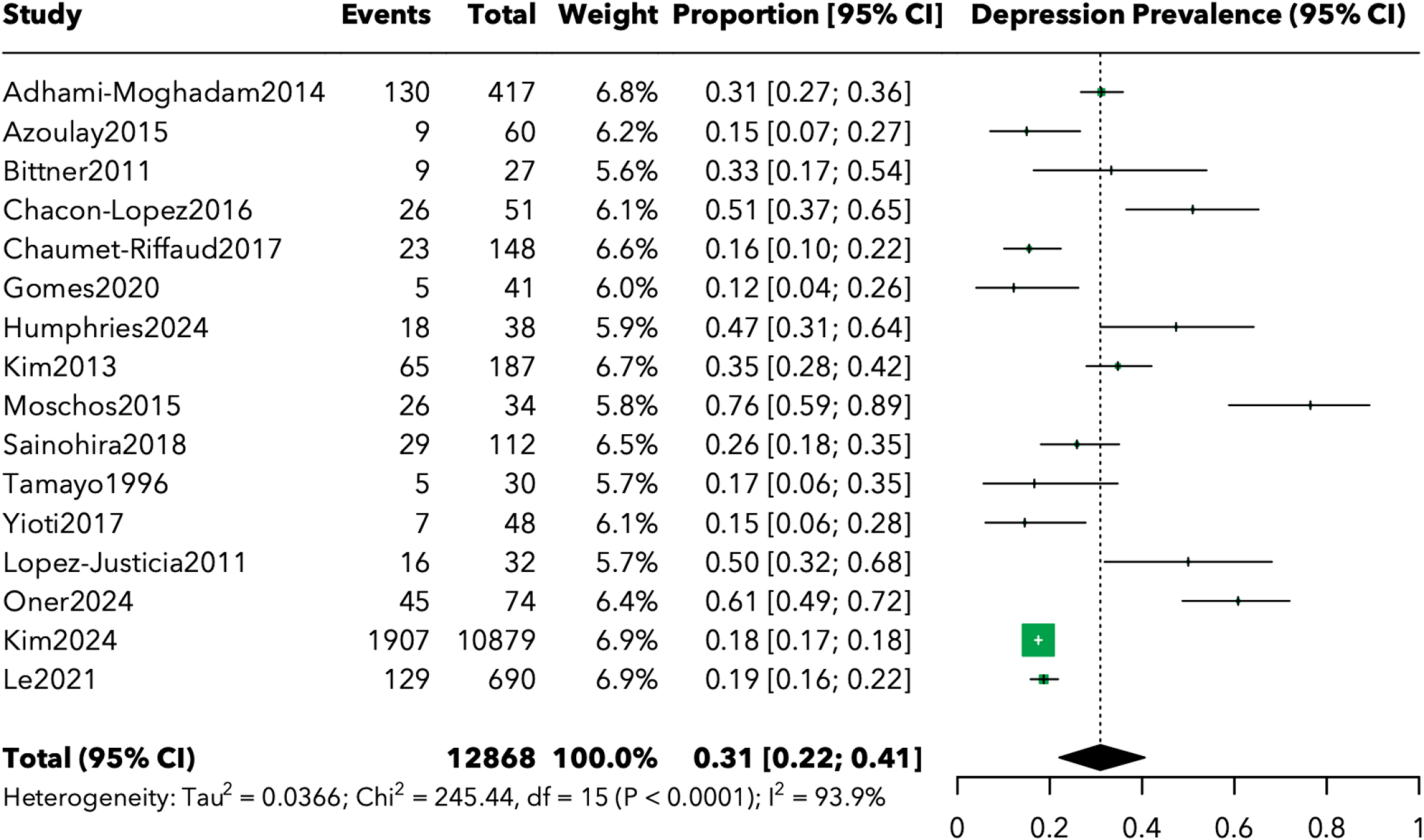
Forest plot of depression prevalence in patients with inherited retinal diseases

The meta-analysis of 16 studies revealed a pooled depression prevalence of 31.0% (95% CI: 22.1%-40.6%) among patients with inherited retinal disorders (Fig. 2). The crude overall prevalence across all studies was 19.0%, with significant variation observed between individual studies ranging from 12.2% [12] to 60.8% [19].

**Fig. 3:**
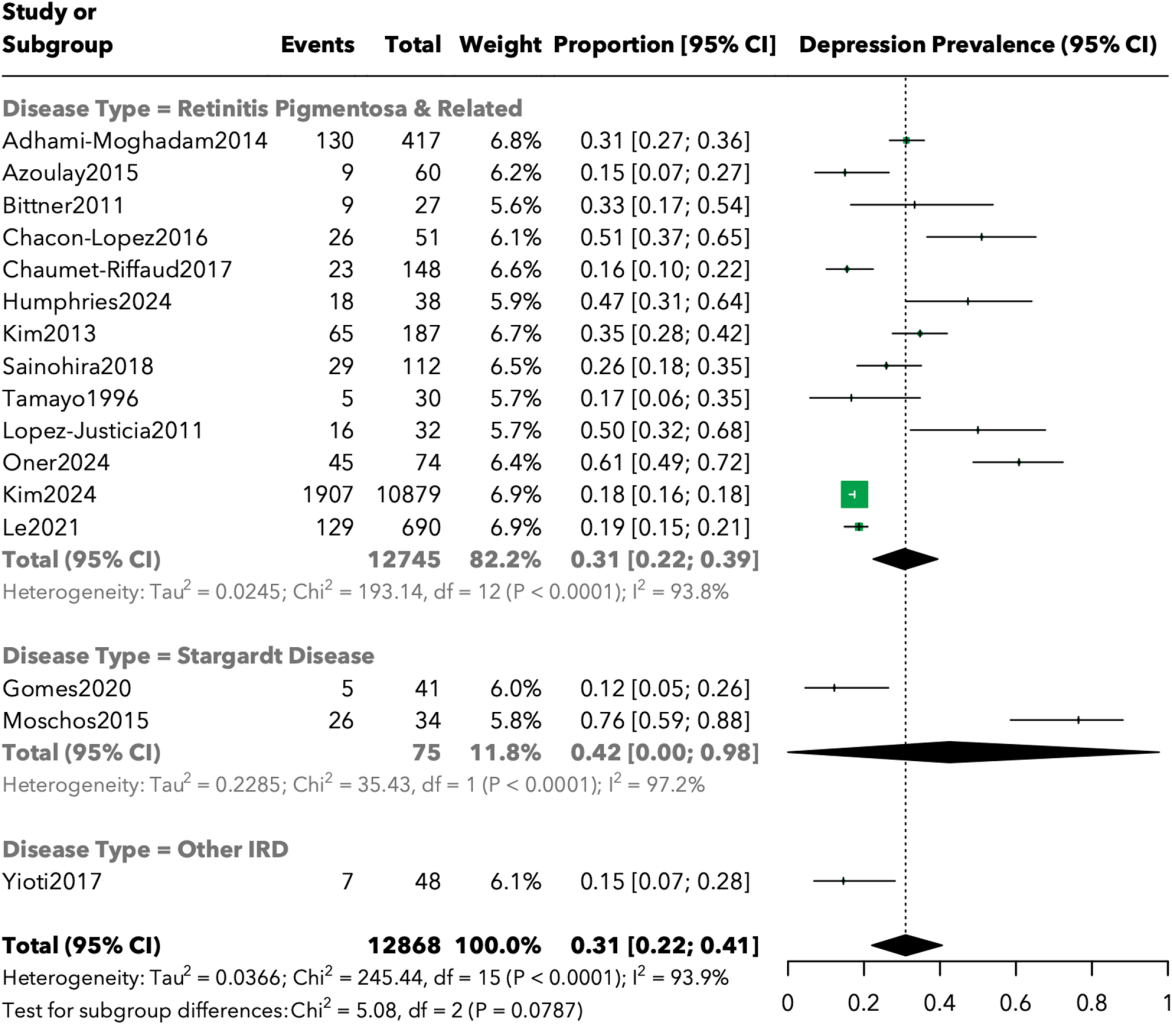
Forest plot of depression prevalence by inherited retinal disease subtype

Subgroup analysis by disease type demonstrated differential depression prevalence patterns (Fig. 3). Patients with retinitis pigmentosa and related conditions (13 studies, *n* = 12,745) showed a pooled prevalence of 30.6% (95% CI: 22.4%-39.4%). Stargardt disease patients (2 studies, *n* = 75) exhibited the highest prevalence at 42.5% (95% CI: 0.0%-97.7%), though this estimate was highly uncertain due to limited data. Other inherited retinal disorders were represented by a single study (*n* = 48) reporting a prevalence of 14.6%. The test for subgroup differences approached statistical significance (Q = 5.08, df = 2, *p* = 0.08), suggesting possible differences between disease categories that warrant further investigation.

Assessment tool analysis revealed varying prevalence rates depending on the measurement approach. Studies using the Beck Depression Inventory reported prevalences ranging from 17.6% [22] to 60.8% [19], while those using HADS reported more conservative estimates ranging from 15.5% [23] to 36.6% [25]. Administrative database studies using ICD diagnostic codes showed intermediate prevalences of 17.5–18.7% [7, 16].

#### Prevalence of anxiety

**Fig. 4:**
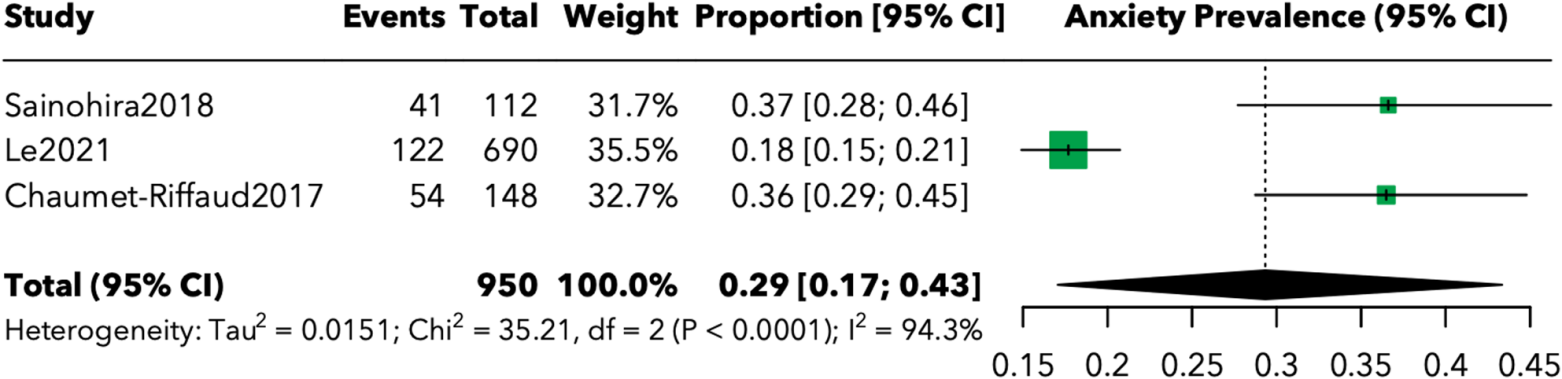
Forest plot of anxiety prevalence in patients with inherited retinal diseases

Analysis of anxiety prevalence (Fig. 4) was limited to 3 studies [7, 23, 25] encompassing 950 participants with RP, yielding a pooled prevalence of 29.3% (95% CI: 17.1%-43.3%). Individual study prevalences ranged from 17.7% in the large administrative database study [7] to 36.5–36.6% in studies using the Hospital Anxiety and Depression Scale [23, 25].

Assessment methodologies varied between the included studies. Two studies employed the HADS-Anxiety subscale [23, 25] with clinical cutoff scores ≥ 8, while the third utilized administrative medical records based on diagnostic codes [7]. This methodological diversity likely contributed to the observed variation in prevalence estimates.

#### Heterogeneity assessment

Both meta-analyses demonstrated substantial between-study heterogeneity. Depression analysis revealed I² = 93.9% (95% CI: 91.5%-95.6%), τ² = 0.0366, and Q = 245.44 (df = 15, *p* < 0.0001). Anxiety analysis showed I² = 94.3% (95% CI: 86.8%-97.6%), τ² = 0.0151, and Q = 35.21 (df = 2, *p* < 0.0001).

Leave-one-out sensitivity analysis for depression (Figure S1) confirmed robust overall findings with stable pooled estimates. Öner 2024 [19] remained influential; its removal changed the pooled estimate from 31.0% to 29.0% and reduced heterogeneity (I² from 93.9% to 92.5%; τ² from 0.0366 to 0.0319), though confidence intervals remained overlapping. Subgroup analysis for retinitis pigmentosa (Figure S2) showed a similar pattern. Excluding Öner 2024 [19] reduced the pooled prevalence from 30.6% to 28.0% (95% CI: 20.9%-35.7%) and heterogeneity (I² = 91.8%, τ² = 0.0171 vs. I² = 93.8%, τ² = 0.0245 in full analysis). Other study removals produced minimal changes (range: 28.2%-32.3%) with persistently high heterogeneity (I² > 93.0%). This pattern highlights the influence of Öner 2024 [19], which reported a moderate-to-severe depression prevalence of 60.8%. Leave-one-out analyses were not performed for Stargardt disease and other IRD subgroups due to insufficient studies (*n* = 2 each).

For anxiety (Figure S3), removing Le 2021 [7] yielded a higher pooled estimate (36.5%, 95% CI: 30.8%-42.5%) with eliminated heterogeneity (I² = 0.0%). The two remaining HADS-based studies [23, 25] showed similar prevalence rates (36.5% and 36.6%), while Le et al.’s administrative database approach yielded 17.7%.

Heterogeneity likely resulted from varied assessment methodologies (validated instruments vs. administrative codes), differing cutoff scores and diagnostic criteria, cultural and healthcare system differences, and varied patient populations (disease severity, duration, demographics).

#### Publication bias

Egger’s test revealed potential publication bias in the depression meta-analysis (t = 3.36, df = 14, *p* = 0.005; bias estimate = 3.15, SE = 0.94), suggesting asymmetry consistent with small-study effects or selective publication of higher prevalence rates.

Funnel plot inspection (Figure S4) showed asymmetrical distribution with a gap in the lower-left portion where smaller studies with lower prevalence rates would be expected. Smaller studies reported higher prevalence rates (right side), while larger studies [7, 16] clustered near the bottom with moderate estimates, supporting statistical evidence of publication bias. This suggests the pooled prevalence estimate of 31.0% may be inflated, though substantial clinical and methodological heterogeneity warrants cautious interpretation. Publication bias assessment for anxiety was not conducted due to insufficient studies (*n* = 3) for reliable testing.

## Discussion

This meta-analysis systematically assessed the prevalence of depression and anxiety among patients with inherited retinal diseases, with particular focus on RP and Stargardt disease. Our findings reveal a pooled depression prevalence of 31.0% (95% CI: 22.1%-40.6%) and anxiety prevalence of 29.3% (95% CI: 17.1%-43.3%) among IRD patients—rates substantially exceeding those observed in the general population, where depression affects approximately 5.7% of adults and anxiety disorders affect 4.05% globally [29, 30] (Table 2). Despite considerable heterogeneity across studies, sensitivity analyses confirmed the robustness of these findings, underscoring a significant psychological burden in this patient population.

**Table 2:** Summary of Pooled Mental Health Outcomes

Outcome	Number of Studies	Total Participants (*n*)	Pooled Prevalence (%)	95% Confidence Interval	I² (%)	τ²	Heterogeneity Test (Q, *p*-value)
**Depression (Overall)**	16	12,868	31.0	22.1–40.6	93.9	0.0366	Q = 245.44, *p* < 0.0001
**Depression by Subgroup**:							
Retinitis Pigmentosa	13	12,745	30.6	22.4–39.4	93.8	0.0245	Q = 193.14, *p* < 0.0001
Stargardt Disease	2	75	42.5	0.0–97.7	95.4	0.1399	Q = 21.79, *p* < 0.0001
Other IRDs*	1	48	14.6	5.8–26.2	--	--	--
Test for subgroup differences							Q = 5.08, df = 2, *p* = 0.08
**Anxiety (Overall)**	3	950	29.3	17.1–43.3	94.3	0.0151	Q = 35.21, *p* < 0.0001

Abbreviations: IRD = Inherited Retinal Disease; I² = measure of heterogeneity (percentage of variation due to heterogeneity); τ² = between-study variance; Q = Cochran’s Q statistic; df = degrees of freedom; CI = Confidence Interval

* Cone-rod Dystorphy, Best’s disease, Usher Syndrom type I & II, Pattern Dystrophy

Our findings align with and extend previous meta-analyses examining mental health outcomes in ophthalmic diseases. Ulhaq et al. (2022) conducted a comprehensive meta-analysis of 95 studies evaluating anxiety symptoms and disorders across various ophthalmic conditions, reporting overall prevalence rates of 31.2% for anxiety symptoms and 19.0% for anxiety disorders [31]. Their subgroup analysis of RP patients revealed an anxiety symptom prevalence of 36.5%, comparable to our estimate of 29.3%. While their work provided valuable broad-spectrum insights, our study advances the field by incorporating depression outcomes, conducting disease-specific and assessment tool-specific analyses, and focusing exclusively on inherited retinal disorders, conditions with unique psychosocial implications due to their genetic nature, early onset, and progressive course. Similarly, Zheng et al. (2017) examined depression prevalence across multiple ophthalmic conditions, including age-related macular degeneration, dry eye disease, glaucoma, and cataracts, reporting an overall pooled prevalence of 25% [32]. Disease-specific estimates ranged from 29% for dry eye disease to 23% for cataracts. Importantly, our observed depression prevalence of 31.0% in IRD patients exceeds these estimates, suggesting that IRDs impose a disproportionately heavy psychological burden compared to other ophthalmic conditions. This difference may reflect the unique challenges of early-onset, progressive, and currently untreatable genetic diseases that profoundly disrupt patients’ life trajectories from youth through adulthood.

We implemented two key methodological refinements during data extraction to enhance construct validity and improve the reliability of our pooled estimates. First, we excluded one study (Stogner, 1980) [28] that assessed “poor personality adjustment” rather than depression measured by validated instruments or standardized diagnostic criteria. This construct, commonly used in the 1980s, does not align with contemporary definitions of depressive disorders and lacks adequate construct validity for inclusion in a modern depression meta-analysis. While this exclusion reduces historical representation, it strengthens the methodological rigor and interpretability of our findings. Second, we extracted only moderate-to-severe depression data (Beck Depression Inventory ≥ 20) from Öner et al. (2024) [19] rather than including all depression levels. This decision maintained consistency with clinically significant thresholds employed across studies, as most BDI-based research uses scores ≥ 20 to indicate clinically meaningful depression. The original study reported 90.5% prevalence when including mild symptoms; our threshold-consistent extraction yielded 60.8%. These refinements reduced heterogeneity (I² from 96.1% to 93.9%) and between-study variance by 36% (τ² from 0.0574 to 0.0366), yielding a more conservative and methodologically homogeneous pooled estimate of 31.0%.

Multiple interconnected factors contribute to the elevated rates of depression and anxiety observed in IRD patients. Depressive symptoms are consistently more prevalent among individuals with chronic diseases compared to the general population [33]. In ophthalmic diseases specifically, systemic pathological mechanisms—including inflammatory processes, circadian rhythm disruption, and metabolic dysregulation—may partially explain the increased depression prevalence [34]. Anxiety symptoms in IRD patients likely arise from feelings of hopelessness, inadequate coping mechanisms in response to progressive and irreversible visual field loss, and uncertainty about disease trajectory [35]. Beyond biological mechanisms, socioeconomic factors play a crucial role in mental health outcomes. The high cost of ongoing medical surveillance, increased healthcare utilization through frequent hospital visits and specialist appointments, and barriers to employment and independent living create substantial psychosocial stress [36, 37]. Additionally, IRD patients frequently experience sleep disturbances, with Ionescu et al. demonstrating that RP patients exhibit disrupted nighttime sleep, excessive daytime sleepiness, and impaired alertness compared to healthy controls [38], factors that may both contribute to and exacerbate depressive symptoms.

The severity of depression in RP correlates significantly with variability in visual field and visual acuity, resulting in decreased physical functioning and intensified depressive symptoms [21]. Furthermore, the emotional impact of IRDs varies considerably based on individual personality characteristics and coping resources [39]. Many RP patients struggle to adapt to progressive visual loss and its cascading effects on vocational prospects, social relationships, and family dynamics [40]. The hereditary nature of these conditions adds another layer of psychological complexity, as patients may experience guilt about potentially transmitting the condition to offspring or witness family members navigating similar challenges.

Visual function parameters, particularly visual acuity (VA) and visual field (VF) loss, likely contribute substantially to the heterogeneity observed in depression prevalence across studies. Bittner et al. (2011) [21] demonstrated that depression severity in RP patients correlates significantly with both VA and VF variability, resulting in decreased physical functioning and intensified depressive symptoms. Similarly, Öner et al. (2024) [19] found that each 0.10 decimal improvement in VA was associated with a 0.85-point decrease in BDI scores, while each 10-decibel reduction in VF mean deviation increased depression scores by 2.5 points. These findings suggest that studies enrolling patients at different disease stages—and consequently different levels of visual function—would be expected to show varying depression prevalence rates. Unfortunately, most included studies in our meta-analysis did not systematically report detailed VA and VF metrics, precluding formal meta-regression analysis to quantify this relationship. Studies that did report visual function data showed considerable variation: some enrolled primarily early-stage patients with preserved central vision [13, 14], while others included advanced cases with legal blindness [19, 25]. This disease stage heterogeneity likely explains a significant portion of the observed between-study variance. Future research should systematically assess and report both VA and VF alongside mental health outcomes, enabling investigation of threshold effects—such as whether depression risk increases sharply when VA falls below specific levels (e.g., < 20/200) or VF constricts beyond certain limits (e.g., < 20 degrees). Such threshold analyses would inform targeted screening protocols and identify high-risk periods during disease progression when mental health support is most critical.

Our subgroup analysis revealed a higher depression prevalence in Stargardt disease patients (42.5%, 95% CI: 0.0%-97.7%) compared to RP patients (33.5%, 95% CI: 21.8%-46.3%). However, this estimate for Stargardt disease must be interpreted with considerable caution, as it was based on only two studies encompassing 75 participants, resulting in extremely wide confidence intervals that span from 0% to nearly 98%. While these preliminary data suggest that Stargardt disease may impose a particularly heavy psychological burden, possibly due to its impact on central vision during formative developmental years, definitive conclusions require substantially more research. The depression prevalence estimate for RP patients, based on 13 studies and 12,745 participants, demonstrates greater precision and aligns closely with previous literature [31]. These findings suggest that while all IRDs carry elevated risk for psychological distress, specific disease characteristics, such as age of onset, pattern of vision loss, and rate of progression, may differentially impact mental health outcomes. The limited data on anxiety outcomes, derived from only three studies encompassing 950 participants, nonetheless revealed a clinically significant pooled prevalence of 29.3%. The high variability in reported anxiety outcomes likely reflects methodological differences, particularly the use of diverse assessment instruments. Two studies employed the Hospital Anxiety and Depression Scale (HADS) with standardized cutoff scores, while the third utilized administrative database codes—approaches that may capture different aspects of anxiety symptomatology and clinical diagnosis. This methodological heterogeneity underscores the critical need for standardized assessment protocols in future research.

The substantial heterogeneity observed in both depression (I² = 93.9%) and anxiety (I² = 94.3%) meta-analyses reflects considerable variability across included studies, though methodological refinements reduced depression heterogeneity from an initial I^2^ of 96.1%. Several factors contributed to this heterogeneity: diverse assessment methodologies ranging from validated psychometric instruments to administrative diagnostic codes; varying cutoff scores and diagnostic criteria; cultural and healthcare system differences across study populations from multiple countries; and heterogeneous patient characteristics including disease severity, duration, and demographic profiles. Leave-one-out sensitivity analyses confirmed the overall robustness of our findings, with pooled estimates remaining relatively stable across iterations. For depression, the Öner 2024 study [19], which reported the highest prevalence of 90.5%, exerted disproportionate influence; its removal reduced the pooled estimate from 31.0% to 29.0% and decreased heterogeneity (I² from 93.9% to 92.5%). Nonetheless, confidence intervals remained overlapping, and the core conclusion of elevated depression prevalence persisted. For anxiety, removing the Le 2021 administrative database study [7] yielded a higher pooled estimate (36.5%) and eliminated heterogeneity (I² = 0%), as the two remaining HADS-based studies [23, 25] reported nearly identical prevalence rates (36.5% and 36.6%). This pattern emphasizes the profound impact of assessment methodology on prevalence estimates and reinforces the importance of standardized measurement approaches.

This study represents the first comprehensive meta-analysis focused exclusively on mental health outcomes in IRD patients. By narrowing the population to IRDs, our findings provide more precise and clinically relevant estimates of depression and anxiety prevalence compared to prior meta-analyses that pooled diverse ophthalmic conditions with varying etiologies, prognoses, and psychosocial implications [31, 32]. Our higher observed depression prevalence (34.1% vs. 25% in Zheng et al.) highlights the particularly heavy psychological burden imposed by genetic, early-onset, progressive retinal diseases. Additionally, our analysis incorporated recent data, rigorous bias assessment, comprehensive heterogeneity analysis, and thorough sensitivity testing—methodological strengths that were limited in previous studies. This disease-specific approach enables more targeted clinical recommendations and identifies specific populations requiring enhanced mental health support.

Our findings carry important implications for clinical practice and healthcare policy. The elevated prevalence of depression and anxiety among IRD patients necessitates systematic integration of mental health screening into routine ophthalmologic care. Healthcare providers managing IRD patients should implement validated screening instruments, such as the Patient Health Questionnaire-9 (PHQ-9) for depression or the Generalized Anxiety Disorder-7 (GAD-7) for anxiety, at regular intervals throughout disease progression. Early identification of psychological distress enables timely intervention, potentially preventing escalation to severe mental illness. Given that congenital and inherited eye diseases profoundly impact quality of life [24], psychological support should be considered an integral component of comprehensive IRD management, delivered alongside ophthalmologic care. This integrated approach might include: establishing streamlined referral pathways to mental health professionals with expertise in chronic illness and visual impairment; developing peer support groups specifically for IRD patients to reduce isolation and share coping strategies; providing psychoeducation about expected disease course and adaptive strategies; offering family counseling to address hereditary aspects and familial impact; and ensuring access to low-vision rehabilitation services that address both functional and psychosocial adaptation. Healthcare systems should consider developing specialized multidisciplinary clinics that coordinate ophthalmologic, genetic counseling, rehabilitation, and mental health services for IRD patients. Such integrated care models have demonstrated effectiveness in other chronic disease populations and could substantially improve outcomes and patient experience for individuals with inherited retinal diseases.

Despite its strengths, this meta-analysis has several limitations that warrant consideration. The substantial heterogeneity in both depression and anxiety analyses limits the generalizability of pooled estimates and suggests that results may not apply uniformly across all IRD populations and clinical contexts. Future research should prioritize standardized assessment tools and diagnostic criteria to enable more direct comparisons and reduce methodological variability.

The limited number of studies examining anxiety in IRD patients (*n* = 3) restricts confidence in anxiety prevalence estimates and precluded formal publication bias assessment. Similarly, only two studies focused specifically on Stargardt disease, yielding highly uncertain prevalence estimates with extremely wide confidence intervals. These gaps highlight critical needs for future research. Evidence of potential publication bias in the depression meta-analysis suggests that the pooled prevalence estimate may be somewhat inflated, as smaller studies with negative or null findings may be underrepresented in the published literature. However, even accounting for potential publication bias, the observed depression prevalence substantially exceeds general population rates, confirming a clinically significant burden. The predominantly cross-sectional design of included studies limits understanding of temporal relationships and causal pathways between IRD progression and mental health deterioration. Longitudinal studies are needed to elucidate how mental health outcomes evolve across disease stages and to identify critical periods for intervention.

Several research priorities emerge from this meta-analysis. First, large-scale, multicenter longitudinal studies should examine the long-term trajectory of mental health outcomes in IRD patients, investigating how depression and anxiety prevalence and severity change with disease progression and identifying predictors of psychological resilience or vulnerability. Second, focused research on underrepresented IRD subtypes, particularly Stargardt disease and rarer inherited retinal dystrophies, is urgently needed to clarify disease-specific mental health risks. Third, intervention studies should evaluate the effectiveness of mental health treatments tailored to IRD patients, including cognitive-behavioral therapy adapted for visual impairment, support group interventions, and integrated care models. Fourth, research should examine the role of genetic counseling in mitigating psychological distress related to hereditary aspects of IRDs and inform development of best practices for family-centered care. Finally, studies should investigate protective factors and successful coping strategies among IRD patients who maintain good mental health despite progressive vision loss, potentially informing preventive interventions.

In conclusion, this meta-analysis demonstrates a substantial mental health burden among patients with IRDs, with pooled prevalence estimates of 31.0% for depression and 29.3% for anxiety, rates significantly exceeding those in the general population. These findings underscore the urgent need for integrated mental health screening and support as a fundamental component of comprehensive IRD management. Healthcare providers should systematically implement validated mental health assessments at diagnosis and throughout disease progression, establish streamlined referral pathways to mental health specialists, and develop multidisciplinary care models addressing both ophthalmologic and psychological needs. Future research should prioritize longitudinal studies examining mental health trajectories across disease stages, expanded investigation of underrepresented IRD subtypes, and evaluation of targeted interventions to improve quality of life in this vulnerable population.

## Supplementary Information

Below is the link to the electronic supplementary material.

Supplementary Material 1

## Data Availability

No datasets were generated or analysed during the current study.
