# Supplementary material for: Mental health outcomes in patients with inherited retinal diseases: a systematic review and meta-analysis
Source: Int J Retina Vitreous. 2026 Feb 20;12:45. doi: 10.1186/s40942-026-00820-7 (PMC13032515; doi:10.1186/s40942-026-00820-7)
Supplement: Supplementary file 1 — Supplementary Material 1 [file 40942_2026_820_MOESM1_ESM.docx]

# Supplementary Materials

##

## Mental Health Outcomes in patients with Inherited Retinal Diseases: A Systematic Review and Meta-Analysis of 12,939 patients

## Supplementary Table S1. Detailed Database Search Strategies

| **Database** | **Results (n)** | **Full search string** |
| --- | --- | --- |
| PubMed | 105 | (“retinitis pigmentosa”[MeSH Terms] OR “retinitis pigmentosa”[Title/Abstract] OR “Stargardt disease”[MeSH Terms] OR “Stargardt disease”[Title/Abstract] OR “fundus flavimaculatus”[Title/Abstract]) AND (“mental health”[MeSH Terms] OR “mental health”[Title/Abstract] OR “depression”[MeSH Terms] OR “depression”[Title/Abstract] OR “anxiety”[MeSH Terms] OR “anxiety”[Title/Abstract] OR “suicidal ideation”[MeSH Terms] OR “suicidal ideation”[Title/Abstract] OR “suicide, attempted”[MeSH Terms] OR “suicide, attempted”[Title/Abstract] OR “mood disorders”[MeSH Terms] OR “mood disorders”[Title/Abstract] OR “psychological distress”[Title/Abstract] OR “psychological morbidity”[Title/Abstract]) |
| Scopus | 238 | (TITLE-ABS-KEY(“retinitis pigmentosa”) OR TITLE-ABS-KEY(“Stargardt disease”) OR TITLE-ABS-KEY(“fundus flavimaculatus”)) AND (TITLE-ABS-KEY(“mental health”) OR TITLE-ABS-KEY(“depression”) OR TITLE-ABS-KEY(“anxiety”) OR TITLE-ABS-KEY(“suicidal ideation”) OR TITLE-ABS-KEY(“suicide, attempted”) OR TITLE-ABS-KEY(“mood disorders”) OR TITLE-ABS-KEY(“psychological distress”) OR TITLE-ABS-KEY(“psychological morbidity”)) |
| Web of Science | 134 | (“retinitis pigmentosa” OR “Stargardt disease” OR “fundus flavimaculatus”) AND (“mental health” OR depression OR anxiety OR “suicidal ideation” OR “suicide attempt*” OR “mood disorder*” OR “psychological distress” OR “psychological morbidity”) |
| Ovid | 106 | (retinitis pigmentosa.mp. or exp Retinitis Pigmentosa/ or Stargardt disease.mp. or exp Stargardt Disease/ or fundus flavimaculatus.ti,ab.) and (mental health.mp. or exp Mental Health/ or depression.mp. or exp Depression/ or anxiety.mp. or exp Anxiety/ or suicidal ideation.mp. or exp Suicidal Ideation/ or suicide, attempted.mp. or exp Suicide, Attempted/ or mood disorders.mp. or exp Mood Disorders/ or psychological distress.ti,ab. or psychological morbidity.ti,ab.) |

## Supplementary Table S2. Newcastle-Ottawa Scale Quality Assessment of Included Studies

| Study | Exposed representation | Ascertainment of exposure | Selection of non-exposed | Outcome was not present at start of study | Comparability of cohorts | Assessment of outcome | Sufficient follow-up | Adequacy of follow-up of cohorts | Total |
| --- | --- | --- | --- | --- | --- | --- | --- | --- | --- |
| **Adhami-Moghadam 2014** | 1 | 1 | 0 | 1 | 0 | 1 | 1 | 1 | **6** |
| **Azoulay 2015** | 1 | 1 | 1 | 1 | 1 | 1 | 1 | 1 | **8** |
| **Bittner 2011** | 1 | 1 | 0 | 1 | 0 | 1 | 1 | 1 | **6** |
| **Chacón-López 2016** | 0 | 1 | 0 | 1 | 0 | 1 | 1 | 1 | **5** |
| **Chaumet-Riffaud 2017** | 1 | 1 | 0 | 1 | 0 | 1 | 1 | 1 | **6** |
| **Gomes 2020** | 1 | 1 | 1 | 1 | 2 | 1 | 1 | 1 | **9** |
| **Humphries 2024** | 0 | 1 | 0 | 1 | 0 | 1 | 1 | 0 | **4** |
| **Kim 2013** | 1 | 1 | 1 | 1 | 2 | 1 | 1 | 1 | **9** |
| **Kim 2024** | 1 | 1 | 0 | 1 | 1 | 1 | 1 | 1 | **7** |
| **Le 2021** | 1 | 1 | 1 | 1 | 1 | 1 | 1 | 1 | **8** |
| **López-Justicia 2010** | 0 | 1 | 1 | 1 | 1 | 1 | 1 | 1 | **7** |
| **Moschos 2015** | 1 | 1 | 1 | 1 | 1 | 1 | 1 | 1 | **8** |
| **Öner 2024** | 1 | 1 | 1 | 1 | 1 | 1 | 1 | 1 | **8** |
| **Sainohira 2018** | 1 | 1 | 0 | 1 | 0 | 1 | 1 | 1 | **6** |
| **Tamayo 1996** | 0 | 1 | 0 | 1 | 0 | 1 | 1 | 1 | **5** |
| **Yioti 2017** | 1 | 1 | 1 | 1 | 2 | 1 | 1 | 1 | **9** |

## Supplementary Figures

### Figure S1. Leave-One-Out Sensitivity Analysis for Depression Prevalence


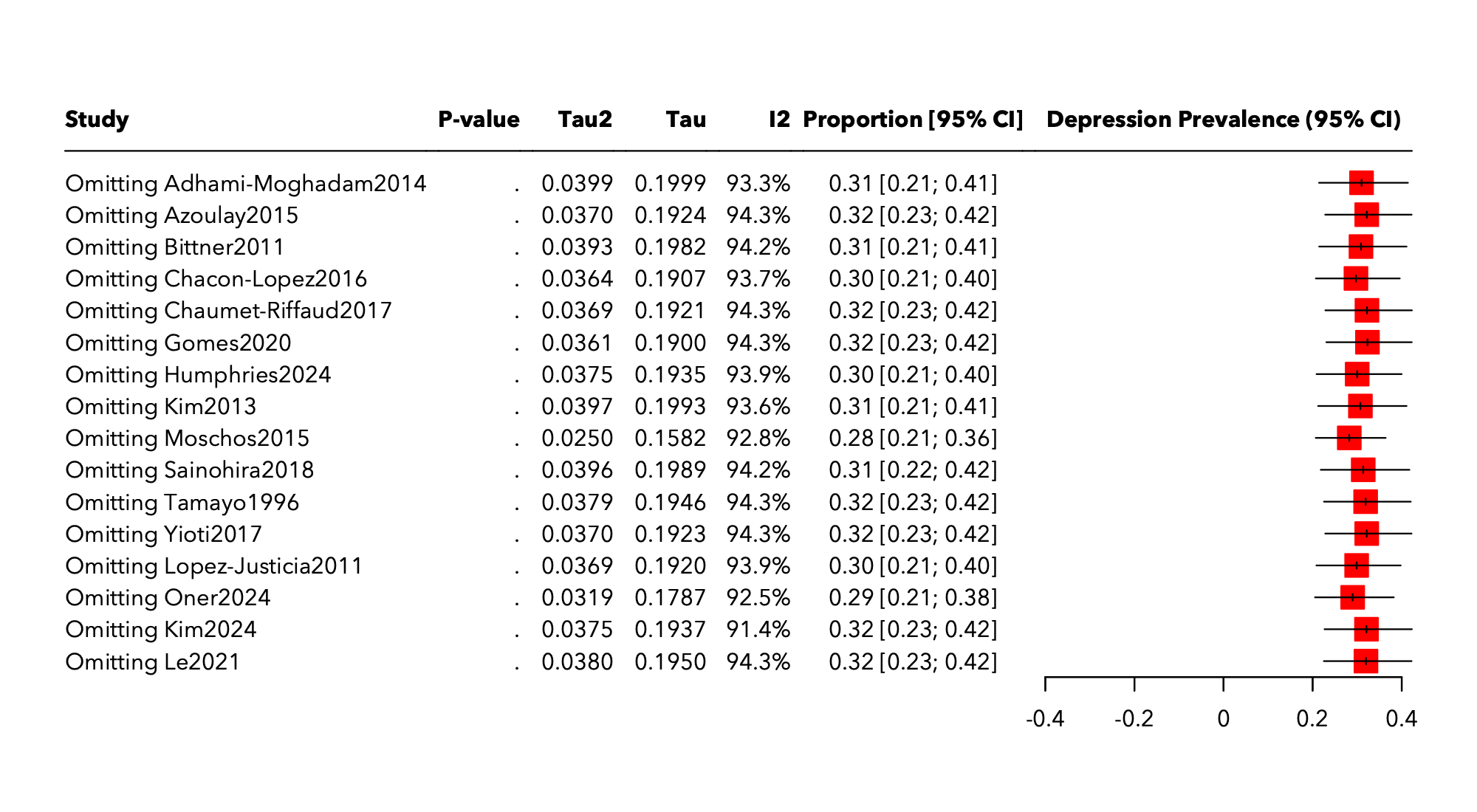


*Forest plot showing the impact of sequentially removing each study on the pooled depression prevalence estimate. Each row represents the meta-analysis result with one study omitted. The plot displays the study excluded, pooled prevalence estimate, 95% confidence interval, and I² statistic for each iteration.*

### Figure S2. Leave-One-Out Sensitivity Analysis for Retinitis Pigmentosa Subgroup (Depression)


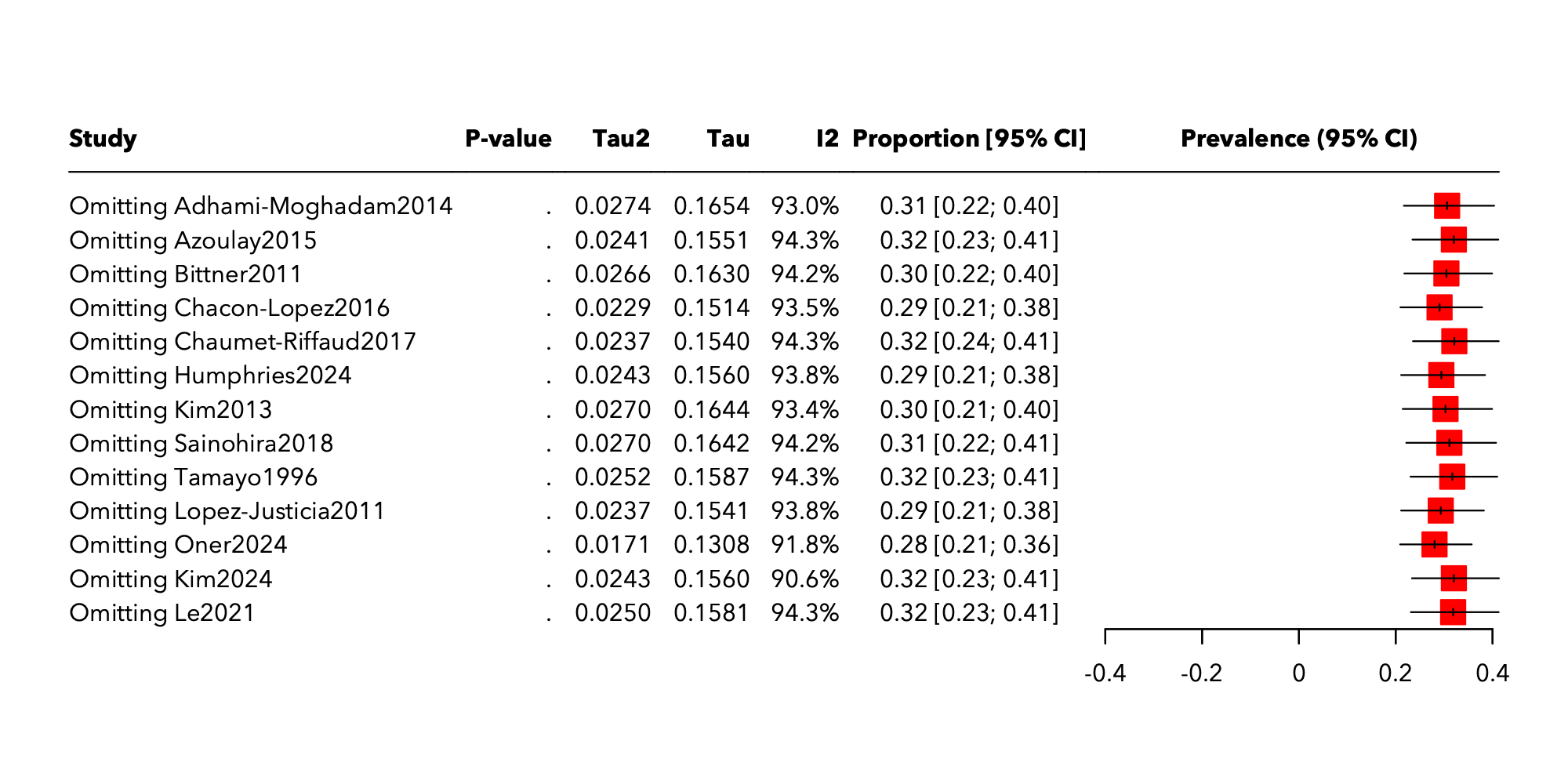


*Forest plot showing leave-one-out sensitivity analysis specifically for the retinitis pigmentosa and related conditions subgroup. Each row shows the pooled prevalence estimate when one study is excluded from the RP subgroup analysis, along with 95% CI and heterogeneity statistics.*

### Figure S3. Leave-One-Out Sensitivity Analysis for Anxiety Prevalence


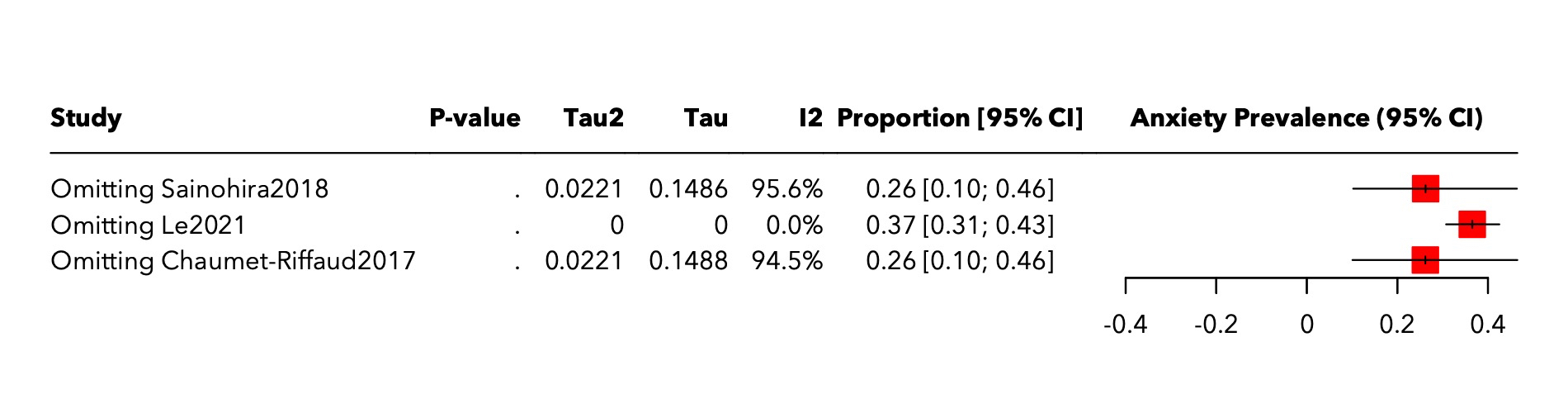


*Forest plot showing the impact of sequentially removing each study on the pooled anxiety prevalence estimate. Given only three studies were included in the anxiety meta-analysis, this figure shows three iterations with corresponding pooled estimates, confidence intervals, and I² statistics.*

### Figure S4. Funnel Plot for Publication Bias Assessment (Depression Meta-Analysis)


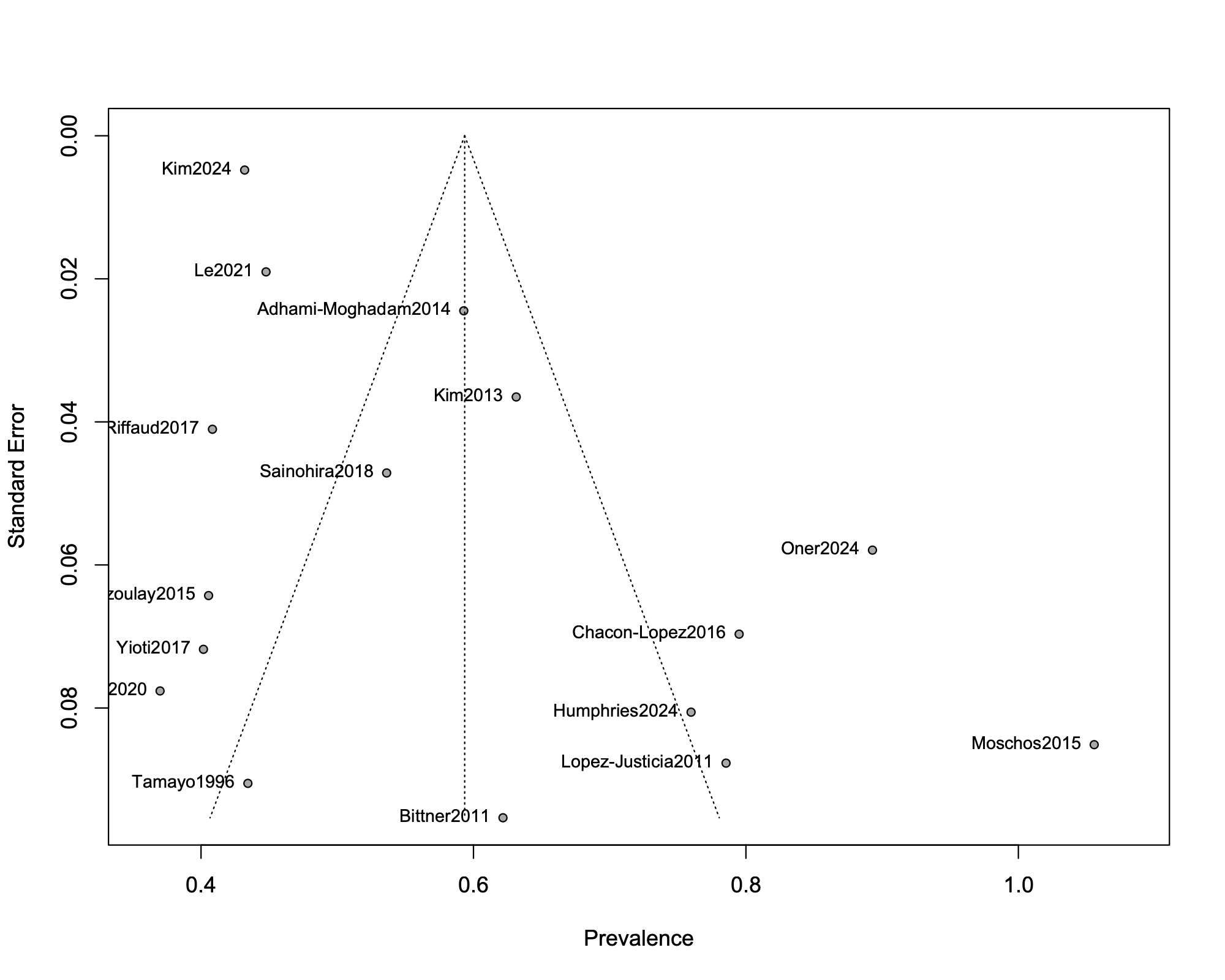


*Funnel plot displaying standard error (y-axis) versus prevalence estimates (x-axis) for the 16 studies included in the depression meta-analysis. The vertical line represents the pooled effect estimate, and the diagonal lines represent the 95% confidence interval boundaries. Asymmetry in the distribution of studies suggests potential publication bias (Egger’s test*(t = 3.36, df = 14, p = 0.005; bias estimate = 3.15, SE = 0.94).
